# Identification and validation of a novel survival prediction model based on the T-cell phenotype in the tumor immune microenvironment and peripheral blood for gastric cancer prognosis

**DOI:** 10.1186/s12967-023-03922-0

**Published:** 2023-02-03

**Authors:** Jing Ma, Jianhui Li, Nan He, Meirui Qian, Yuanyuan Lu, Xin Wang, Kaichun Wu

**Affiliations:** 1 Department of Gastroenterology, Tangdu Hospital, The Air Force Military Medical University, Xi’an, 710032 Shaanxi China; 2 State Key Laboratory of Cancer Biology and Institute of Digestive Diseases, The Air Force Military Medical University, Xi’an, China; 3 Department of Infectious Diseases, Tangdu Hospital, The Air Force Military Medical University, Xi’an, China; 4 National Translational Science Center for Molecular Medicine, The Air Force Military Medical University, Xi’an, China; 5 State Key Laboratory of Cancer Biology and Xijing Hospital of Digestive Diseases, The Air Force Military Medical University, Xi’an, 710032 Shaanxi China

**Keywords:** Inhibitory molecule, Tumor immune microenvironment, Peripheral blood lymphocytes, Survival prediction model, Gastric cancer

## Abstract

**Background:**

The correlation and difference in T-cell phenotypes between peripheral blood lymphocytes (PBLs) and the tumor immune microenvironment (TIME) in patients with gastric cancer (GC) is not clear. We aimed to characterize the phenotypes of CD8^+^ T cells in tumor infiltrating lymphocytes (TILs) and PBLs in patients with different outcomes and to establish a useful survival prediction model.

**Methods:**

Multiplex immunofluorescence staining and flow cytometry were used to detect the expression of inhibitory molecules (IMs) and active markers (AMs) in CD8^+^TILs and PBLs, respectively. The role of these parameters in the 3-year prognosis was assessed by receiver operating characteristic analysis. Then, we divided patients into two TIME clusters (TIME-A/B) and two PBL clusters (PBL-A/B) by unsupervised hierarchical clustering based on the results of multivariate analysis, and used the Kaplan–Meier method to analyze the difference in prognosis between each group. Finally, we constructed and compared three survival prediction models based on Cox regression analysis, and further validated the efficiency and accuracy in the internal and external cohorts.

**Results:**

The percentage of PD-1^+^CD8^+^TILs, TIM-3^+^CD8^+^TILs, PD-L1^+^CD8^+^TILs, and PD-L1^+^CD8^+^PBLs and the density of PD-L1^+^CD8^+^TILs were independent risk factors, while the percentage of TIM-3^+^CD8^+^PBLs was an independent protective factor. The patients in the TIME-B group showed a worse 3-year overall survival (OS) (HR: 3.256, 95% CI 1.318–8.043, P = 0.006), with a higher density of PD-L1^+^CD8^+^TILs (P < 0.001) and percentage of PD-1^+^CD8^+^TILs (P = 0.017) and PD-L1^+^CD8^+^TILs (P < 0.001) compared to the TIME-A group. The patients in the PBL-B group showed higher positivity for PD-L1^+^CD8^+^PBLs (P = 0.042), LAG-3^+^CD8^+^PBLs (P < 0.001), TIM-3^+^CD8^+^PBLs (P = 0.003), PD-L1^+^CD4^+^PBLs (P = 0.001), and LAG-3^+^CD4^+^PBLs (P < 0.001) and poorer 3-year OS (HR: 0.124, 95% CI 0.017–0.929, P = 0.015) than those in the PBL-A group. In our three survival prediction models, Model 3, which was based on the percentage of TIM-3^+^CD8^+^PBLs, PD-L1^+^CD8^+^TILs and PD-1^+^CD8^+^TILs, showed the best sensitivity (0.950, 0.914), specificity (0.852, 0.857) and accuracy (κ = 0.787, P < 0.001; κ = 0.771, P < 0.001) in the internal and external cohorts, respectively.

**Conclusion:**

We established a comprehensive and robust survival prediction model based on the T-cell phenotype in the TIME and PBLs for GC prognosis.

**Supplementary Information:**

The online version contains supplementary material available at 10.1186/s12967-023-03922-0.

## Background

During the progression of cancer, the genetic and cellular alteration of tumor cells provides neoantigens, differentiation antigens or cancer testis antigens to the immune system to generate T-cell responses that recognize tumor cells [1]. Although the presence of lymphocytes in the tumor microenvironment (TME), in particular CD8^+^T cells, is correlated with good prognosis in several solid tumors, these CD8^+^ tumor infiltrating lymphocytes (TILs) fail to effectively eliminate tumor cells and infrequently provide protective immunity [2]. The prognostic value of CD8^+^TILs in gastric cancer (GC) is controversial, which is not only related to the differences among samples in different studies [3, 4], but also more likely to be related to the functional status of CD8^+^TILs. Persistent stimulation with tumor antigens results in a state of T-cell dysfunction or exhaustion, which can upregulate several coinhibitory molecules, such as PD-1, CTLA-4, TIM-3 and LAG-3 [5]. In non-small cell lung cancer and renal clear cell cancer, the high expression of inhibitory molecules (IMs) on CD8^+^T cells was related to poor prognosis [6, 7]. Multiple immunohistochemistry (IHC) and flow cytometry are common investigatory tools to explore the immune context in the TME. The former provides objective quantitative data on immune cells, but its antibody characteristics and staining scheme are limited. Flow cytometry is performed using a cell suspension causing the histological structure to be lost and sometimes showing inconsistent results compared with IHC [8].

Circulating immune cell concentrations, including granulocytes, monocytes, lymphocytes, and myeloid-derived suppressor cells (MDSCs), change during tumor progression and antitumor therapy [9]. Compared with T cells in TILs, it is more convenient to investigate the alterations in peripheral blood T-cell subset distribution and functional status because peripheral blood is easy to obtain and detect by flow cytometry. Several studies have shown that the percentage of peripheral blood CD4^+^/CD8^+^ effector memory T (T_EM_) cells and IMs expression on T cells were higher in patients with GC than in heathy donors (HDs). Moreover, these abnormities were associated with tumor invasion, differentiation and lymphatic metastasis [10–12].

To date, numerous gene/molecular markers of tissue or blood have been applied to identify the progression and prognosis of GC. However, it is not sufficient to predict the prognosis using just one marker. In this study, we analyzed IMs (PD-1, PD-L1 and TIM-3) expression on CD8^+^TILs, investigated the phenotypes and functional status of T cells in peripheral blood lymphocytes (PBLs) and further explored their relationships with prognosis.

## Methods

### Patients and tissues

We collected both formalin-fixed paraffin-embedded (FFPE) tissue specimens and peripheral blood samples from 47 consecutive patients with GC from June 1st 2016 to August 1st 2016 in Xijing Hospital, as well as 48 peripheral blood samples from HDs. All patients were diagnosed with primary GC and received surgeries with no prior cancer treatment, including chemotherapy, radiotherapy, targeted therapy and immune therapy. Written informed consent was obtained from all participants. This study was executed under ethical approval from the First Affiliated Hospital of the Fourth Military Medical University (No. KY20192088-F-1).

### Multiplex immunofluorescence staining

We evaluated H&E (Hematoxylin–eosin)-stained slides of each case and selected the FFPE block with the largest area of viable tumor cells and lowest area of necrotic tissue. Normal tonsil tissue was used as a positive control. Briefly, 3 to 5-μm-thick sections were deparaffinized and rehydrated, and antigen retrieval was performed (EDTA pH 9.0 for PD-L1 and TIM-3, and citrate pH 6.0 for PD-1). After blocking the endogenous peroxidase activity with 3% H_2_O_2_ and nonspecific antigens with normal goat serum, the primary antibody cocktails, which included CD8 (C8/144B, mouse IgG1,CST, 1:100) + PD-1 (EH33, mouse IgG2a, CST, 1:200), CD8 (C8/144B, mouse IgG1,CST, 1:100) + PD-L1 (SP142, rabbit IgG, Spring Bioscience Corp, 1:100) and CD8 (C8/144B, mouse IgG1,CST, 1:100) + TIM-3 (D5D5R™, rabbit IgG, CST, 1:200) were incubated overnight at 4 ℃. Then, sections were incubated with corresponding secondary antibodies, including goat anti-mouse IgG1 cross-adsorbed secondary antibody, Alexa Fluor 594 (Invitrogen, A-21125) + goat anti-mouse IgG2a cross-adsorbed secondary antibody, Alexa Fluor 647 (Invitrogen, A-21241), goat anti-mouse IgG1 cross-adsorbed secondary antibody, Alexa Fluor 633 (Invitrogen, A-21126) + goat anti-rabbit IgG (H + L) highly cross-adsorbed secondary antibody, and Alexa Fluor 594 (Invitrogen, A-11037). Each secondary antibody was diluted 1:1000. All sections were covered using Fluoroshield containing 4′,6-diamidino-2-phenylindole (DAPI) for 10 min at RT. Each stained slide was captured using a digital slide scanner (3DHISTECH, Budapest, Hungary) and analyzed by connected QuantCenter software (3DHISTECH, Budapest, Hungary). For multiplex immunofluorescence, filters for Texas Red and Cy5 were used for fluorescence excitation with an external light source. All components were integrated and controlled using QuantCenter software. This analysis platform was used to quantify the number and the density of single- and double-positive cells in whole tissue sections.

### IHC

Consecutive FFPE sections were cut from each specimen. Primary antibodies, including anti-CD3 (D7A6E™, rabbit IgG, CST, 1:200), anti-45RO (UCHL1, mouse IgG2a, CST, 1:400), anti-CD8 (C8/144B, mouse IgG1, CST, 1:200), anti-CD11c (D3V1E, rabbit IgG, CST, 1:400), anti-Foxp3 (D2W83™, rabbit IgG, CST, 1:100), anti-CD20 (L26, mouse IgG2a, DAKO), anti-CD68 (KP1, mouse IgG1, DAKO) and anti-CD163 (10D6, mouse IgG1, Novus, 1:100) were incubated at 4 ℃ overnight. Then, the sections were incubated with secondary antibodies. Normal tonsil tissue was used as a positive control. Each stained slide was captured using a digital slide scanner (3DHISTECH, Budapest, Hungary) and analyzed by connected QuantCenter software (3DHISTECH, Budapest, Hungary). For IHC, the total number and density of cells expressing targeted proteins were quantified.

### Flow cytometry

PBMCs were isolated from whole blood by a human peripheral blood lymphocyte separation tube (DKW-LST-25050SK, Dakewe, China) and stored at -80 ℃. We designed five staining panels to assess the T-cell subsets, activation markers (AMs) and IMs expression, including Panel 1—anti-human CD45 (HI30, FITC), CD4 (RPA-T4, PE), CD8 (RPA-T8, APC), CD69 (FN50, PE/Cy7), CD38 (HIT2, Percp/Cy5.5) and HLA-DR (G46-6, APC/H7); Panel 2—anti human CD45 (2D1, Percp), CD4 (OKT4, PE/Cy7), CD8 (SK1, APC/Cy7), CD45RA (HI100, APC), CCR7 (G043H7, FITC) and CD27 (O323, PE); Panel 3—anti-human CD45 (HI30, FITC), CD4 (RPA-T4, PE), CD8 (RPA-T8, APC), BTLA (MIH26, PE/Cy7), LAG-3 (11C3C65, Percp/Cy5.5) and TIM-3 (F38-2E2, APC/Fire750); Panel 4—anti-human CD45 (HI30, FITC), CD4 (RPA-T4, PE), CD8 (RPA-T8, APC), CTLA-4 (29E.2A3, PE/Cy7), PD-L1 (L3D10, Percp/Cy5.5) and PD-1 (EH12.2H7, APC/Cy7); Panel 5—anti human CD45 (2D1, Percp), CD4 (OKT4, Percp/Cy5.5), CD127 (HIL-7R-M21, AF647) and CD25 (M-A251, PE). Samples were recovered at 37 ℃ and incubated with the antibodies for 30 min at RT. The fluorescence minus one (FMO) control was performed for each investigated antigen in each panel to identify positive and negative cells. Samples were detected using BD FACSAria II flow cytometry and data were analyzed by FlowJo v.10.0 (Tree Star, Franklin Lakes, NJ, USA). The gating strategy is presented in Additional file 2: Fig. S2.

### Magnetic Luminex cytokine assay

The frozen HDs and patient PBMCs were thawed and 300,000 cells were plated into each well at a concentration of 3 × 10^5^ cells/mL in a 24-well plate. For the group that was to be activated, we added 25 μL/mL anti-CD3/CD28 (ImmunoCult™ human CD3/CD28 T-cell activator, Stemcell). After 72 h, the supernatant was collected from each well, centrifuged at 1000×*g* for 5 min and stored at − 80 ℃. We used a magnetic Luminex assay to detect the levels of IFN-γ (No. of bead: 30), IL-2 (No. of bead: 19), IL-5 (No. of bead: 22) and IL-13 (No. of bead: 34) in the supernatant. The procedures were performed according to the manufacturer’s instructions.

### Models and cohorts

When developing prediction models for time-to-event outcomes, we calculated the sample size to ensure 10 events per candidate predictor parameter (EPP). However, the actual required sample size depends on multiple factors, including the number of events relative to the number of candidate predictor parameters, the total number of participants, the incidence in the study population and the expected predictive performance of the model [13]. Models of less than 10 EPP may provide acceptable confidence interval (CI) coverage and bias, but the results need to be interpreted with caution [14]. In this study, we established three survival prediction models based on 47 patients, designated as the internal cohort. Model 1 and Model 2 were constructed based on TIME and PBL characteristics, respectively, while Model 3 was based on both characteristics. We screened three candidate predictor parameters by multivariate Cox regression analysis in each model, meaning the EPP was 7 in all three models (20 deaths corresponding to three candidate predictor parameters). The regression coefficient (β) of each parameter in each model was also calculated by the multivariate Cox regression analysis. An ROC curve was used to determine the cut-off value, which was the corresponding value of the maximal Youden index (sensitivity + specificity − 1). Each candidate predictor parameter was transformed into dichotomies based on the cut-off values. The 3-year survival rate of patients (S(t)) was calculated in the internal cohort. The formula was 1 − S(t)^exp ($\sum_{i=0}^{p}\mathrm{\beta iXi}$ − $\sum_{i=0}^{p}\mathrm{\beta i\overline{X}i}$). The Kappa–Cohen method was used to assess the concordance between the predicted results and the actual results.

To validate the effectiveness of the three models, an external cohort was set up which incorporated 70 consecutive patients with primary GC admitted from October 1st 2016 to December 1st 2016 in Xijing Hospital. Thirty-five patients died over three years in the external cohort, meaning that the EPP was 12 (35 deaths corresponding to three parameters).

### Statistical analysis

Data are shown as the mean ± SD or SEM. We used an independent—sample T test to compare the distribution of T-cell subsets and the positivity of AMs and IMs in PBLs between GC and HDs. The Kruskal–Wallis test was used to compare the positivity between different clusters, and paired-sample T tests were used to compare the expression of PD-1, PD-L1, and TIM-3 on CD8^+^T cells between TILs and PBLs. An ROC curve was used to determine the cut-off value of TILs and PBLs in survival prediction. The unsupervised clustering of patients was performed by hierarchical methods using Euclidian distance. The associations of patient clusters (TIME A/B and PBL-A/B), PD-1^+^CD8^+^TILs (%), PD-L1^+^CD8^+^TILs (%), TIM-3^+^CD8^+^TILs (%), the density of PD-L1^+^CD8^+^TILs and prognosis were evaluated using the Kaplan–Meier method. All p values were based on two-sided tests, and values less than 0.05 was considered statistically significant. SPSS 21.0 (SPSS Inc., Chicago, IL, USA) and GraphPad Prism v6.0 (GraphPad Software, Inc., San Diego, CA, USA) were used to analyze the data and prepare figures.

## Results

### Unsupervised clustering of CD8^+^TILs defined two clusters of GC patients with distinct immune characteristics and different prognosis

**Fig. 1:**
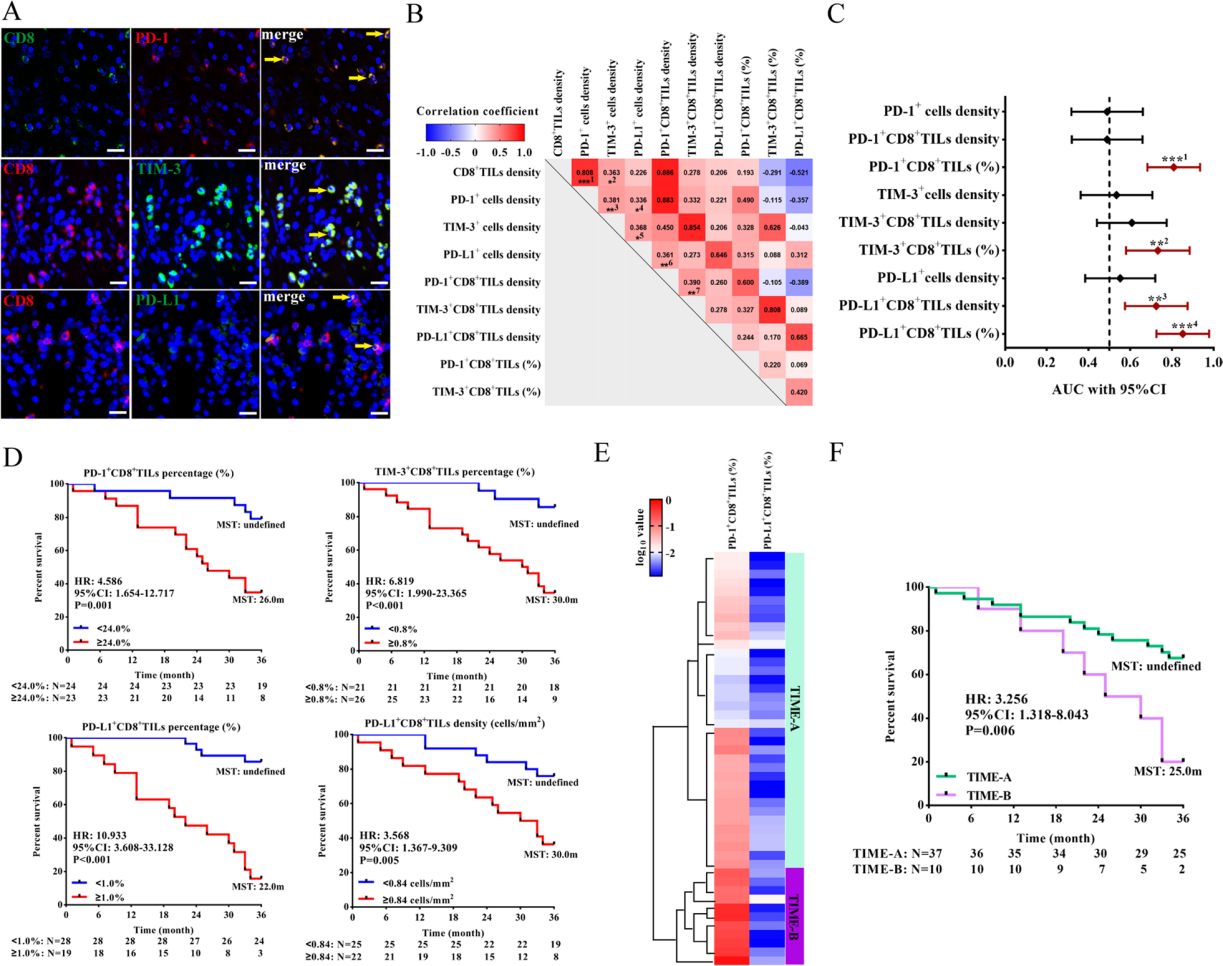
The prognosis and characteristics of immune markers in two clusters of GC patients defined by unsupervised hierarchical clustering analysis. **A** Multiplex immunofluorescence staining of PD-1, PD-L1 and TIM-3 expression on CD8^+^TILs. The yellow arrow indicates double-positive cells. Bar: 20 μm. **B** Correlation analysis among CD8^+^TILs, IMs (PD-1, PD-L1 and TIM-3) and IMs^+^CD8^+^TILs. ^***1^: P < 0.001, ^*2^: P = 0.012, ^*3^: P = 0.008, ^*4^: P = 0.021, ^*5^: P = 0.011, ^*6^: P = 0.013, ^**7^: P = 0.007. **C** ROC curves and AUCs with 95% CI were used to evaluate the accuracy of IM expression, IM^+^CD8^+^ TIL density and proportion in predicting 3-year prognosis (n = 47). ^***1^: P < 0.001, ^**2^: P = 0.007, ^**3^: P = 0.009, ^***4^: P < 0.001. **D** Survival analysis of the different levels of IMs^+^CD8^+^TIL infiltration at the corresponding cut-offs (24.0% for PD-1^+^CD8^+^TILs, 0.8% for TIM-3^+^CD8^+^TILs, 0.84 cells/mm^2^ and 1.0% for PD-L1^+^CD8^+^TILs). (E) The heatmap of the percentage of PD-1^+^CD8^+^TILs and PD-L1^+^CD8^+^TILs in patients with TIME-A (n = 37) and TIME-B (n = 10) defined by unsupervised hierarchical clustering. (F) Survival analysis of the two clusters. TIL: tumor infiltrating lymphocytes, IMs: inhibitory molecules, ROC: receiver operating characteristic, AUC: area under the curve. *P < 0.05, **P < 0.01, ***P < 0.001

We analyzed the expression of three IMs (PD-1, TIM-3 and PD-L1) in CD8^+^TILs by multiple immunofluorescence staining (Fig. 1A). The median density and percentage of PD-1^+^CD8^+^TILs, TIM-3^+^CD8^+^TILs and PD-L1^+^CD8^+^TILs were 18.91 cells/mm^2^ (23.50%), 0.44 cells/mm^2^ (0.50%) and 0.47 cells/mm^2^ (0.78%), respectively. The density of PD-1-expressing (ρ = 0.808, P < 0.001) and TIM-3-expressing (ρ = 0.363, P = 0.012) cells was correlated with the density of CD8^+^TILs (Fig. 1B). There was no difference in the expression of IM and CD8 among different T stages, N stages and histological types (Additional file 1: Fig. S1). To evaluate the role of the three IMs and their coexpression with CD8 on T cells in prognosis, we calculated the area under the ROC curve of each parameter. The density of PD-L1^+^CD8^+^TILs (AUC: 0.725, 95% CI 0.575–0.875, P = 0.009) and the percentage of PD-1^+^CD8^+^TILs (AUC: 0.808, 95% CI 0.682–0.935, P < 0.001), TIM-3^+^CD8^+^TILs (AUC: 0.732, 95% CI 0.580–0.885, P = 0.007) and PD-L1^+^CD8^+^TILs (AUC: 0.852, 95% CI 0.726–0.978, P < 0.001) could predict 3-year OS, with the cut-offs of 0.84 cells/mm^2^, 24.0%, 0.8%, and 1.0%, respectively (Fig. 1C and D). However, only the percentage of PD-1^+^CD8^+^TILs (HR: 5.857, 95% CI 1.915–17.919, P = 0.002) and PD-L1^+^CD8^+^TILs (HR: 12.410, 95% CI 3.885–39.639, P < 0.001) were independent risk factors for prognosis in multivariate analysis (Table 1).

**Table 1:** Univariate and multivariate Cox regression analysis of the characteristics in TIME and 3-year overall survival in the internal cohort

Characteristic	Univariate analysis		Multivariate analysis	
	HR (95% CI)	P value	HR (95% CI)	P value
AJCC stage	1.364 (1.052–1.769)	0.019		
N status	1.706 (1.229–2.368)	0.001	1.453 (1.022–2.065)	0.037
Lauren’s classification	0.958(0.648–1.416)	0.829		
IMs expressed on CD8^+^T cells in TIME				
CD8^+^TILs density	0.995 (0.990–1.000)	0.064		
PD-1 density	1.000 (0.994–1.007)	0.944		
PD-1^+^CD8^+^TILs density	1.001 (0.992–1.010)	0.862		
PD-1^+^CD8^+^TILs (%)	4.586 (1.654–12.717)	0.003	5.857 (1.915–17.919)	0.002
TIM-3 density	1.006 (0.993–1.019)	0.368		
TIM-3^+^CD8^+^TILs density	1.098 (0.949–1.270)	0.208		
TIM-3^+^CD8^+^TILs (%)	6.819 (1.990–23.365)	0.002		
PD-L1 density	0.999 (0.995–1.004)	0.818		
PD-L1^+^CD8^+^TILs density	1.744 (1.183–2.572)	0.005		
PD-L1^+^CD8^+^TILs (%)	10.933(3.608–33.128)	< 0.001	12.410 (3.885–39.639)	< 0.001
Immune cells in TIME				
CD3^+^ TILs density	0.999 (0.997–1.001)	0.492		
CD8^+^ TILs density	0.995 (0.990–1.000)	0.064		
CD8 to CD3 ratio	0.031 (0.002–0.521)	0.016	0.128 (0.029–0.566)	0.007
CD45RO^+^ TILs density	0.999 (0.997–1.001)	0.307		
CD11c^+^ cells density	0.929 (0.810–1.065)	0.290		
CD20^+^ cells density	0.981 (0.898–1.071)	0.665		
CD68^+^ cells density	1.033 (0.972–1.097)	0.295		
CD163^+^ cells density	1.085 (0.985–1.196)	0.099		
CD163 to CD68 ratio	3.215 (0.747–13.839)	0.117		
Foxp3^+^ Tregs density	0.538 (0.273–1.061)	0.074		

Then, we categorized patients into two clusters by unsupervised hierarchical clustering, named TIME-A and TIME-B (Fig. 1E). The clinicopathological characteristics of the two clusters were shown in Additional file 6: Table S1. The density of PD-1^+^CD8^+^TILs (P = 0.049), TIM-3^+^cells (P = 0.019), TIM-3^+^CD8^+^TILs (P = 0.011), PD-L1^+^cells (P < 0.001) and PD-L1^+^CD8^+^TILs (P < 0.001), and the percentage of PD-1^+^CD8^+^TILs (P = 0.017) and PD-L1^+^CD8^+^TILs (P < 0.001) in TIME-B were higher than those in TIME-A. Patients in the TIME-B group exhibited a shorter 3-year OS than those in the TIME-A group (HR: 3.256, 95% CI 1.318–8.043, P = 0.006, Fig. 1F).

### Patients with different peripheral blood T-cell characteristics showed different prognoses

**Fig. 2:**
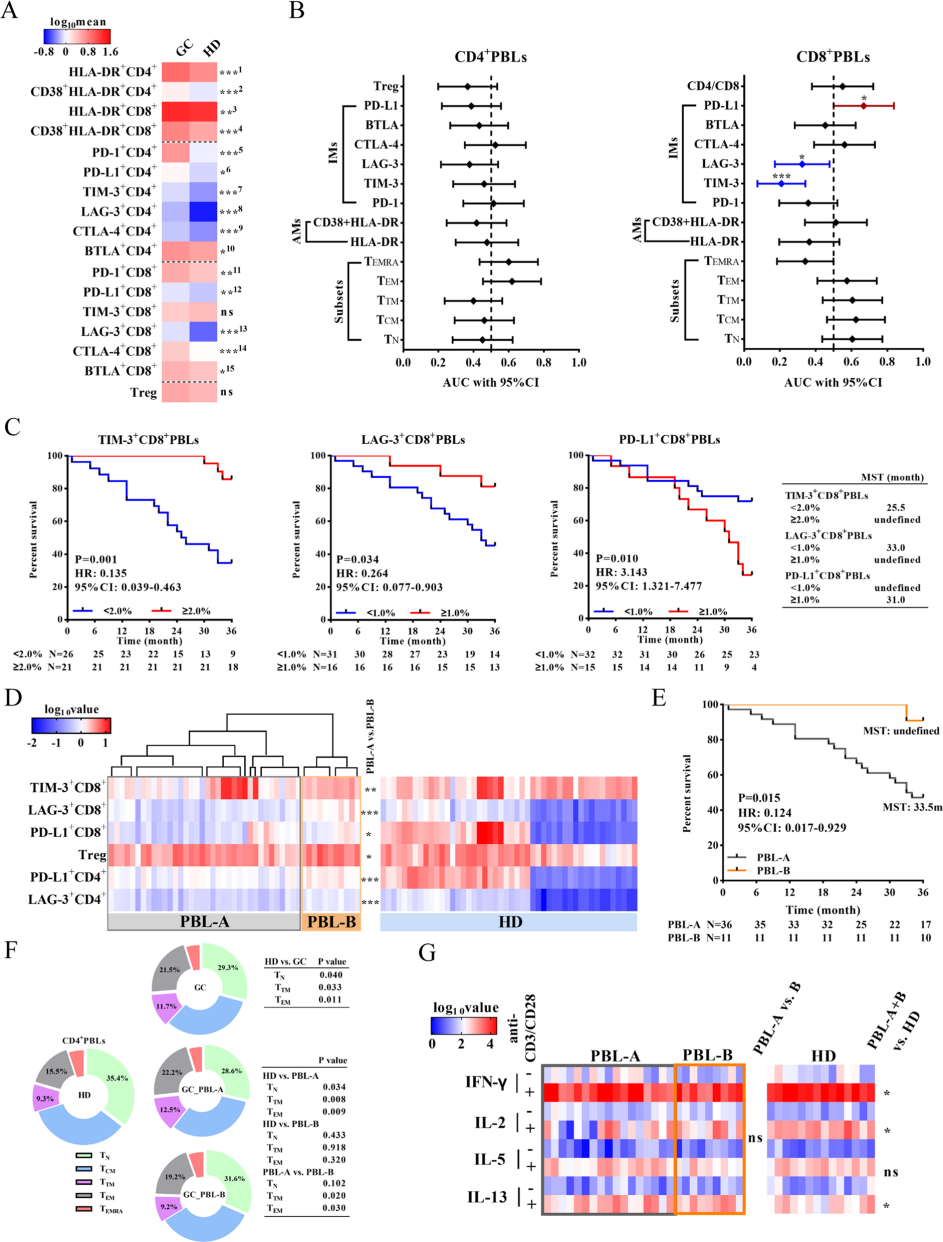
The phenotypic characteristics of peripheral blood T cells. **A** The level of AMs (CD38 and HLA-DR) expression, IMs (PD-1, TIM-3, LAG-3, CTLA-4, BTLA and PD-L1) expression and the percentage of Tregs among peripheral T cells in the HD (n = 48) and GC (n = 47) groups. The difference is displayed on the right. ^***1^: P < 0.001, ^***2^: P = 0.001, ^**3^: P = 0.007, ^***4^: P < 0.001, ^***5^: P < 0.001, ^*6^: P = 0.012, ^***7^: P < 0.001, ^***8^: P < 0.001, ^***9^: P = 0.001,^*10^: P = 0.026, ^**11^: P = 0.004, ^**12^: P = 0.008, ^***13^: P = 0.001, ^***14^: P < 0.001, ^*15^: P = 0.015. **B** ROC curves and AUCs with 95% CI were used to evaluate the accuracy of peripheral CD4^+^/CD8^+^T-cell subset distribution, and AM^+^CD4^+^/CD8^+^ and IMs^+^CD4^+^/CD8^+^ proportions in predicting the prognosis of GC (n = 47). **C** Survival analysis of different levels of TIM-3^+^CD8^+^PBLs, LAG-3^+^CD8^+^PBLs and PD-L1^+^CD8^+^PBLs. **D** Based on the peripheral T-cell phenotype, PBL-A (n = 36) and PBL-B (n = 11) were grouped by unsupervised hierarchical clustering. The significant difference is displayed on the right of the heatmap. **E** Survival analysis of patients in PBL-A and PBL-B. **F** Distribution of peripheral CD4^+^T-cell subsets of HDs (n = 48), and patients in the GC (n = 47), PBL-A (n = 36) and PBL-B (n = 11) groups. **G** The cytokine secretion levels before and after anti-CD3/CD28 stimulation in vitro. *P < 0.05, **P < 0.01, ***P < 0.001

First, we compared the differences in AMs and IMs expression in PBLs between patients with GC and HDs by flow cytometry (Additional file 2: Fig. S2 and Additional file 3: Fig. S3). The positivity of AMs (CD38 and HLA-DR) and IMs (PD-1, TIM-3, CTLA-4, BTLA and LAG-3) on CD4^+^ and CD8^+^T cells in the peripheral blood of patients with GC was significantly higher than that in the peripheral blood of HDs (Fig. 2A). The ratio of CD4 to CD8 cells in GC was lower than that in HDs (0.93 ± 0.07 vs. 1.18 ± 0.08, P = 0.025). According to ROC curves of AMs, IMs and T subsets in PBL (Fig. 2B), the positivity of TIM-3^+^CD8^+^PBLs (AUC: 0.208, 95% CI 0.074–0.342, P = 0.001), LAG-3^+^CD8^+^PBLs (AUC: 0.324, 95% CI 0.172–0.477, P = 0.041) and PD-L1^+^CD8^+^PBLs (AUC: 0.669, 95% CI 0.501–0.837, P = 0.049) could predict 3-year OS, with the cut-offs of 2.0%, 2.0%, and 1.0%, respectively (Fig. 2B and C). Multivariate analysis showed that the positivity of TIM-3^+^CD8^+^PBLs (HR: 0.134, 95% CI 0.037–0.485, P = 0.002) and PD-L1^+^CD8^+^PBLs (HR: 4.309, 95% CI 1.738–10.682, P = 0.002) were independent protective and risk factors for GC, respectively (Table 2). There was no difference in the distribution of T-cell subsets or AMs and IMs expression on T cells among different T stages, N stages and histological types (Additional file 4: Fig. S4).

**Table 2:** Univariate and multivariate Cox regression analysis of the characteristics in peripheral blood and 3-year overall survival in the internal cohort

	Univariate analysis		Multivariate analysis	
	HR (95% CI)	P	HR (95% CI)	P
AJCC stage	1.364 (1.052–1.769)	0.019		
N status	1.706 (1.229–2.368)	0.001	1.610 (1.116–2.322)	0.011
CD4 to CD8 ratio	1.350 (0.544–3.351)	0.518		
Subsets				
CD4^+^T_N_	0.990 (0.958–1.023)	0.552		
CD4^+^T_CM_	0.983 (0.935–1.034)	0.513		
CD4^+^T_TM_	0.908 (0.812–1.016)	0.093		
CD4^+^T_EM_	1.050 (1.005–1.097)	0.028		
CD4^+^T_EMRA_	1.021 (0.954–1.093)	0.546		
CD8^+^T_N_	1.010 (0.972–1.049)	0.610		
CD8^+^T_CM_	1.115 (1.006–1.236)	0.038		
CD8^+^T_TM_	1.026 (0.946–1.112)	0.540		
CD8^+^T_EM_	1.020 (0.981–1.060)	0.318		
CD8^+^T_EMRA_	0.980 (0.954–1.006)	0.134		
Treg	0.706 (0.485–1.026)	0.068		
Activation				
HLA-DR^+^CD4^+^T	1.121 (0.983–1.279)	0.088		
CD38^+^HLA-DR^+^CD4^+^T	0.726 (0.302–1.744)	0.474		
HLA-DR^+^CD8^+^T	0.951 (0.862–1.048)	0.311		
CD38^+^HLA-DR^+^CD8^+^T	1.062 (0.908–1.242)	0.453		
IMs				
PD-1^+^CD4^+^T	1.067 (0.868–1.312)	0.537		
PD-L1^+^CD4^+^T	0.305 (0.083–1.116)	0.073		
TIM-3^+^CD4^+^T	1.170 (0.481–2.848)	0.729		
CTLA-4^+^CD4^+^T	1.581 (0.308–8.114)	0.583		
LAG-3^+^CD4^+^T	0.180 (0.020–10,631)	0.127		
BTLA^+^CD4^+^T	0.902 (0.778–1.045)	0.170		
PD-1^+^CD8^+^T	0.911 (0.791–1.049)	0.196		
PD-L1^+^CD8^+^T	3.143(1.321–7.477)	0.010	4.309 (1.738–10.682)	0.002
TIM-3^+^CD8^+^T	0.135 (0.039–0.463)	0.001	0.134 (0.037–0.485)	0.002
CTLA-4^+^CD8^+^T	1.343 (0.848–2.127)	0.209		
LAG-3^+^CD8^+^T	0.264 (0.077–0.903)	0.034		
BTLA^+^CD8^+^T	0.967 (0.762–1.227)	0.783		

Then, we divided patients into two groups (PBL-A and PBL-B) with distinct phenotypic characteristics by hierarchical unsupervised clustering (Fig. 2D). Compared to PBL-A, the major feature of patients in PBL-B was higher positivity of PD-L1^+^CD8^+^ (P = 0.042), LAG-3^+^CD8^+^ (P < 0.001), TIM-3^+^CD8^+^ (P = 0.003), PD-L1^+^CD4^+^ (P = 0.001), and LAG-3^+^CD4^+^ (P < 0.001), and a higher percentage of Treg (P = 0.021). The 3-year OS of patients in the PBL-B group was significantly longer than that of patients in the PBL-A group (HR: 0.124, 95% CI 0.017–0.929, P = 0.015, Fig. 2E).

In general, the proportion of CD4^+^T_N_ cells decreased (P = 0.040), while the proportions of CD4^+^T_TM_ (P = 0.033) and CD4^+^T_EM_ (P = 0.011) cells increased in the GC group compared to the HD group (Fig. 2F). The distribution of CD4^+^PBLs in the PBL-B group was similar to that in the HD group (Fig. 2F). Furthermore, we evaluated the secretion levels of IFN-γ, IL-2, IL-5 and IL-13 after anti-CD3/CD28 stimulation in vitro. Lower levels of IFN-γ (P = 0.040) and IL-2 (P = 0.021), and a higher level of IL-13 (P = 0.030) were detected in GC patients than in HDs. However, there was no significant difference in cytokine secretion between the PBL-A and PBL-B groups (Fig. 2G). The clinicopathological characteristics of the PBL-A and PBL-B groups are shown in Additional file 6: Table S2.

### IM expression on CD8^+^PBLs was correlated with that on CD8^+^TILs

**Fig. 3:**
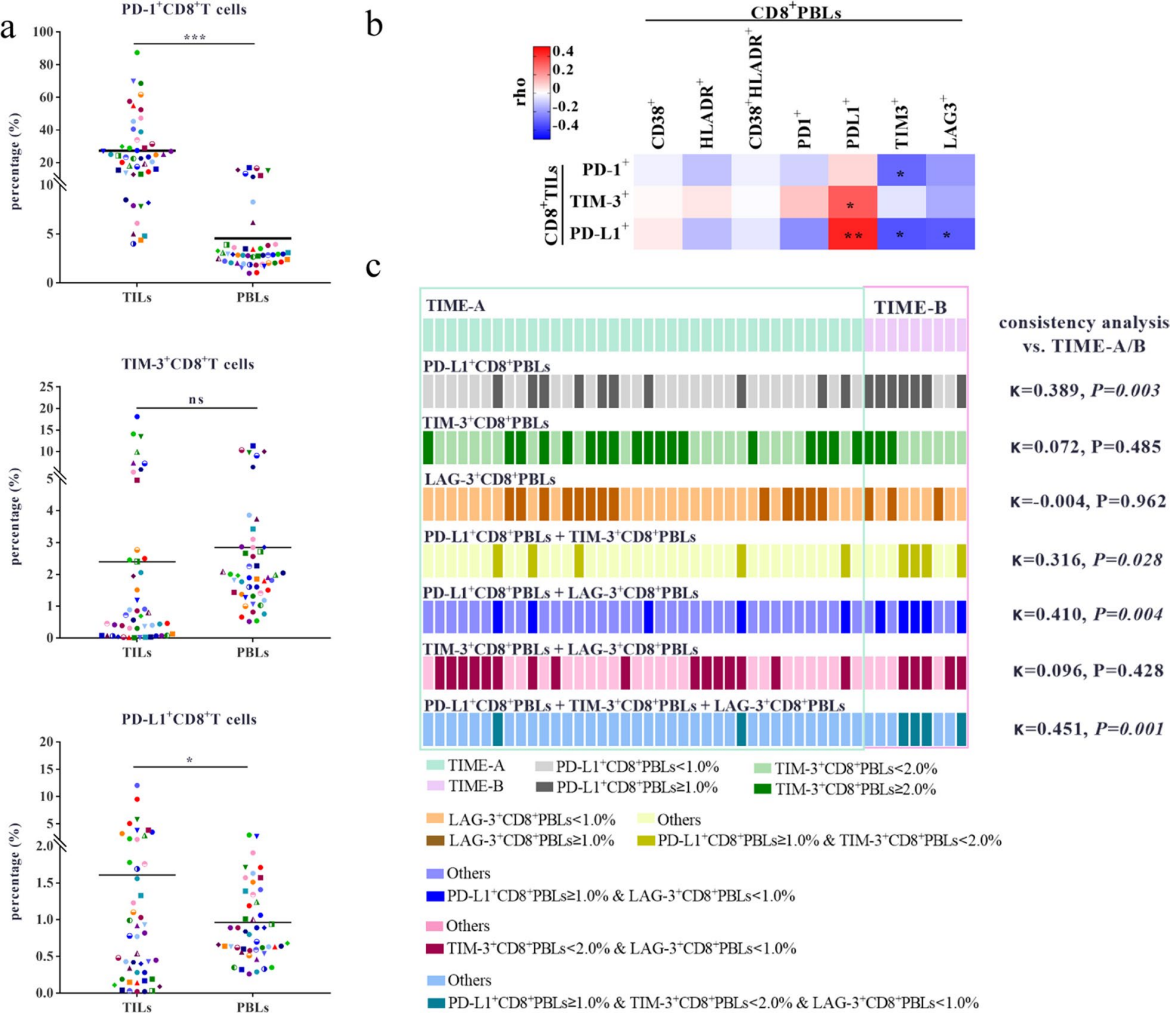
Correlation and difference analysis of IMs expression between PBLs and TILs. **A** The difference and correlation analysis of PD-1, TIM-3 and PD-L1 expression between CD8^+^PBLs and CD8^+^TILs. The expression of PD-1, TIM-3 and PD-L1 on CD8^+^T cells in TILs and corresponding PBLs (n = 47). The dots of the same shape and color denote the same patients. **B** Correlation analysis between IMs^+^CD8^+^TILs, and AMs^+^CD8^+^PBLs and IMs^+^CD8^+^PBLs in 47 patients. The analysis was performed using samples from the same patients. **C** The consistency analysis between two clusters of TIME and the grouping results of single/pairwise/simultaneous combination of three parameters, including the percentage of PD-L1^+^CD8^+^PBLs, TIM-3^+^CD8^+^PBLs and LAG-3^+^CD8^+^PBLs. *P < 0.05, **P < 0.01

The percentages of PD-1^+^CD8^+^T cells (P < 0.001) and PD-L1^+^CD8^+^T cells (P = 0.046) were lower in PBLs than in TILs (Fig. 3A). There was no significant difference (P = 0.493, Fig. 3A) or consistency (κ = 0.067, P = 0.227) in the expression of TIM-3 between the CD8^+^TILs and CD8^+^PBLs. The percentage of PD-L1^+^CD8^+^T cells was moderately consistent between TILs and PBLs at the 1% cut-off (κ = 0.484, P = 0.001). Correlation analysis showed that the percentage of PD-L1^+^CD8^+^TILs was moderately positively correlated with the percentage of PD-L1^+^CD8^+^PBLs (ρ = 0.432, P = 0.002), and negatively correlated with the percentage of TIM-3^+^CD8^+^PBLs (ρ = – 0.339, P = 0.020) and LAG-3^+^CD8^+^PBLs (ρ = – 0.323, P = 0.027, Fig. 3B). The percentage of PD-1^+^CD8^+^TILs was positively correlated with the percentage of TIM-3^+^CD8^+^PBLs (ρ = – 0.298, P = 0.042).

We further explored whether PBL parameters could profile the two clusters of the TIME. Single, pairwise and simultaneous combination of the following three parameters, including PD-L1^+^CD8^+^PBLs percentage, TIM-3^+^CD8^+^PBLs percentage and LAG-3^+^CD8^+^PBLs percentage, could not group the patients of TIME-A/B effectively (Fig. 3C). Although the result of grouping by the percentage of PD-L1^+^CD8^+^PBLs, TIM-3^+^CD8^+^PBLs and LAG-3^+^CD8^+^PBLs (κ = 0.451, P = 0.001) measured simultaneously was most consistent with those of TIME-A/B, only 44.4% of patients in TIME-B were identified.

### A comprehensive model incorporating both TILs and PBLs effectively predicted the 3-year overall survival

**Fig. 4:**
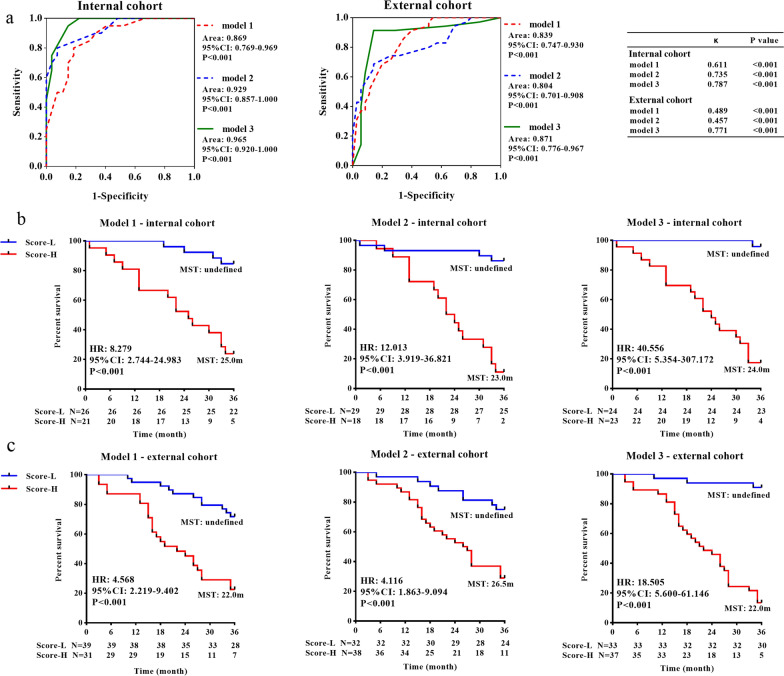
The prognostic predictive effect of the three survival prediction models. **A** ROC curves of three survival prediction models for GC in predicting 3-year survival in the internal cohort and external cohort. The 3-year survival analysis of patients with different scores (Score-L vs. Score-H) in three models in the internal (**B**) and external cohorts (**C**)

According to the ROC curve analysis (Additional file 5: Fig. S5 and Additional file 6: Table S3), the single parameters of TIME and PBLs could not effectively predict the 3-year overall survival. Therefore, we established three survival prediction models based on different combinations of clinical pathological characteristics, TIME and PBL parameters (Table 3). Model 1 was based on N stage, PD-1^+^CD8^+^TILs (%) and PD-L1^+^CD8^+^TILs (%), and Model 2 was based on N stage, PD-L1^+^CD8^+^PBLs (%) and TIM-3^+^CD8^+^PBLs (%). Model 3 was based on TIM-3^+^CD8^+^PBL (%), PD-1^+^CD8^+^TIL (%) and PD-L1^+^CD8^+^TILs (%) (Additional file 6: Table S4). To confirm the prognostic value of the three models, we further applied them to the internal and external cohorts (Fig. 4A, the clinicopathological characteristics of the two cohorts are shown in Additional file 6: Table S5). According to the ROC analysis, the sensitivity/specificity of the Models 1, 2 and 3 in the internal cohort at each calculated cut-off point were 0.800/0.815, 0.800/0.926 and 0.950/0.852, respectively. The sensitivity/specificity of the Models 1, 2 and 3 in the external cohort were 0.686/0.800, 0.743/0.686 and 0.914/0.857, respectively. Both in the internal (Fig. 4B) and external cohorts (Fig. 4C), patients with high scores in Model 1, 2 and 3 showed poorer prognoses than those with low scores (P < 0.001).

**Table 3:** Three survival prediction models

	Regression coefficient^∀^	Formula	Cut-off^&^
Model 1			
N stage	0.374	1 − S(t)^*^^exp(w(t)^$^ − 2.2480^#^)	0.589
PD-1^+^CD8^+^TILs (%)	1.768		
PD-L1^+^CD8^+^TILs (%)	2.518		
Model 2			
N stage	0.476	1 − S(t)^exp(w(t) + 0.5475^#^)	0.694
PD-L1^+^CD8^+^PBLs (%)	1.461		
TIM-3^+^CD8^+^PBLs (%)	− 2.011		
Model 3			
TIM-3^+^CD8^+^PBLs (%)	− 2.006	1 − S(t)^exp(w(t) − 1.2014^#^)	0.508
PD-L1^+^CD8^+^TILs (%)	2.821		
PD-1^+^CD8^+^TILs (%)	1.960		

^∀^ Regression coefficient was determined by multivariate Cox regression analysis

^*^S(t) was defined as three-year survival rate. We calculated the survival rate to be 0.574

^$^w(t) = $$\sum_{i=0}^{p}\mathrm{\beta iXi}$$ βi = regression coefficient for each parameter, Xi = The detection value or classification value of the corresponding parameter

^#^The constant was calculated according $\sum_{i=0}^{p}\mathrm{\beta i \overline{X}i}$

^&^Cut-off point was the corresponding value of the maximal Youden index. Youden index = sensitivity + specificity − 1

To evaluate the accuracy of the three models, we performed consistency analysis between the predicted and actual prognoses. The predicted results of Model 3 (κ = 0.787, P < 0.001 in the internal cohort; κ = 0.771, P < 0.001 in the external cohort) were strongly consistent with the actual results. For Model 1, a strong (κ = 0.611, P < 0.001) and a moderate (κ = 0.486, P < 0.001) consistency was shown in the internal and external cohorts, respectively. Model 2 exhibited moderate consistency in the internal (κ = 0.735, P < 0.001) and external cohorts (κ = 0.457, P < 0.001).

## Discussion

Our study demonstrated that patients with GC were characterized by a trend of differentiation from CD4^+^T_N_ to CD4^+^T_TM_/T_EM_ in PBLs, and a higher AMs and IMs expression on T cells in PBLs compared with HDs. Although the IMs expression in CD8^+^TILs was different from that in CD8^+^PBLs, the percentages of PD-L1^+^CD8^+^PBLs and PD-L1^+^CD8^+^TILs were moderately consistent at the 1% cut-off. According to the unsupervised hierarchical clustering analysis, we divided patients into two clusters (TIME-A and TIME-B) and two groups (PBL-A and PBL-B) based on the CD8^+^TILs and CD8^+^PBLs phenotypic features, respectively. The three-year survival rate of patients in the TIME-B and PBL-A groups was lower. Moreover, we constructed a survival prediction model in patients with GC, which included three parameters (the percentage of TIM-3^+^CD8^+^PBLs, PD-L1^+^CD8^+^TILs and PD-1^+^CD8^+^TILs) and showed a high sensitivity, specificity and consistency in both internal and external cohorts.

The analysis of immune cells from peripheral blood could be used to stratify cancer patients to predict survival and immunotherapy response, although its value in studying tumor–immune interactions is limited [15, 16]. In general, patients with GC showed distinct "high IM expression" and "chronic activation" PBL phenotypes compared to HDs. Further analysis found that patients with favorable prognosis (PBL-A) were characterized by high expression of IM on CD8^+^PBLs, which was inconsistent with studies in other tumors [7, 17]. The overexpression of IM (PD-1, TIM-3, LAG-3, CTLA-4 and BTLA) was described as markers of "T cell exhaustion" [18]. However, we did not find a functional difference in T cells between the PBL-A and B groups in the cytokine secretion assay. This fact may be because we analyzed peripheral T cells as a whole and did not conduct cytokine secretion tests after sorting IM^+^PBLs and IM^−^PBLs. Recent studies have suggested that IMs expression on CD8^+^T cells is tightly related to the status of cell differentiation and activation [19, 20]. We considered that high IM expression might be more likely to reflect the activation of T cells (correlation analysis: R = 0.503, P = 0.001 between PD-1^+^CD8^+^ and HLA-DR^+^CD8^+^; R = 0.534, P < 0.001 between TIM-3^+^CD8^+^ and HLA-DR^+^CD8^+^).

PD-L1, as the ligand of PD-1, is also expressed on multiple hematopoietic cells, including activated T cells, DCs, macrophages and mesenchymal stem cells. We previously found that the expression of PD-L1 on CD8^+^TILs was correlated with a worse prognosis [21]. The PD-L1 status in circulating white blood cells correlated with PD-L1 expression on immune cells in tumor tissue [22]. Our study showed that the expression of PD-L1 between CD8^+^PBLs and CD8^+^TILs was moderately consistent, and the prognostic value showed a similar trend at the same threshold. Evaluating PD-L1 expression on CD8^+^TILs and patient prognosis by detecting peripheral PD-L1^+^CD8^+^T cells, which are highly accessible, would be feasible. However, the results should be interpreted with caution because of the different detection techniques between TILs and PBLs. In future studies, we will use flow cytometry to analyze IM and AM expression in fresh tumor tissues and corresponding peripheral blood samples.

Compared to peripheral blood, the immune context inside tumors is more likely to reflect the interactions between tumor cells and immune cells. TILs coexpressing IM reflected both functional activation of tumor/tumor associated antigens and T-cell dysfunction. Patients in Cluster B were characterized as having a "high inhibitory and exhausted" TIME with a higher percentage of IMs^+^CD8^+^. Cancer patients with higher frequencies of PD-1^high^CD8^+^TILs showed a worse disease-free survival and OS, a higher simultaneous expression of multiple IMs in CD8^+^TILs, and a more susceptible response to combined immune therapies [6, 23, 24]. Notably, our results emphasized the percentage of PD-1^+^CD8^+^TILs rather than the density of PD-1^+^CD8^+^TILs as a poor prognostic factor and survival prediction, which might be attributable to the strong correlation between the density of CD8^+^TILs and PD-1^+^CD8^+^TILs. Moreover, we observed that the proportion of PD-1-expressing CD8^+^TILs was significantly higher than that in CD8^+^PBLs from GC and HDs. PD-1-expressing CD8^+^T-cell infiltration in the tumor context may reflect both CD8^+^T-cell activation and exhaustion, while PD-1-expressing CD8^+^PBLs may reflect tumor-associated and nontumor-associated antigen experience [25]. Another major inhibitory co-receptor TIM-3, is mainly expressed in IFN-γ producing CD4^+^ and CD8^+^T cells, especially in terminally differentiated effector Th1 cells [26]. Our study found that the survival-predicting significance of the TIM-3-expressing CD8^+^T-cell proportion in TILs was completely opposite to that in PBLs. We speculated that the high proportion of TIM-3^+^CD8^+^TILs reflected T-cell exhaustion, while the elevated proportion of TIM-3^+^CD8^+^PBLs might reflect systematic immune activation.

Undoubtedly, larger sample sizes lead to the development of more robust models. However, in some cases, a large sample size is not always available, especially when the sample includes difficult-to-achieve parameters. A simulation study found that the worst instances of each problem (confidence interval coverage less than 93 percent, type I error greater than 7 percent, and relative bias greater than 15 percent) were not severe with 5–9 EPP and were usually comparable to those with 10–16 EPP [14]. In our study, Model 3 showed higher sensitivity and better consistency than Model 1 and 2. Moreover, ROC analysis demonstrated that Model 3 had parallel sensitivity (0.950 vs. 0.914), specificity (0.852 vs. 0.857) and accuracy (0.787 vs. 0.771) between the internal and external cohorts. Although Model 3 was derived from a relatively small sample, it was well validated in a larger sample, indicating that the model was acceptable.

Recently, several signatures based on the immune/tumor-related gene expression and clinical-molecular characteristics were used to evaluate and predict prognosis [27–30]. Our Model 3 incorporated the characteristics of systematic and local CD8^+^T cells, and was validated to predict 3-year survival effectively in both the internal and external cohorts. The convenience detection methods of the three parameters made this model much simpler to use in clinical practice. However, a larger sample cohort is still needed to validate the efficiency of the survival prediction model.

## Supplementary Information

**Additional file 1: Figure S1.** The difference of the density of CD8^+^TILs, PD-1^+^cells, PD-L1^+^cells, TIM-3^+^cells, PD-1^+^CD8^+^TILs, PD-L1^+^CD8^+^TILs and TIM-3^+^CD8^+^TILs, and the difference of the percentage of PD-1^+^CD8^+^TILs, PD-L1^+^CD8^+^TILs and TIM-3^+^CD8^+^TILs were analyzed based on the T stage, N stage and Lauren's classification.

**Additional file 2: Figure S2.** The analysis diagram of AMs, IMs, and Treg in PBLs.

**Additional file 3: Figure S3.** The analysis diagram of T cell subsets in PBLs.

**Additional file 4: Figure S4.** The T cell subsets distribution, AMs and IMs expression on T cells in PBLs were analyzed based on the T stage, N stage and Lauren's classification.

**Additional file 5: Figure S5.** The analysis of the predicted survival value by ROC curves.

**Additional file 6: Table S1.** The clinical-pathological characteristics of TIME-A and TIME-B. **Table S2.** The clinical pathological characteristics of PBL-A and PBL-B. **Table S3.** Sensitivity and specificity of TIME and PBLs parameters in predicting prognosis. **Table S4.** Multivariate Cox regression analysis for the candidate parameters of model 3 in the internal cohort. **Table S5.** The clinical pathological characters of patients.

## Data Availability

The datasets used and/or analyzed during the current study are available from the corresponding author on reasonable request.

## Abbreviations

AMs: Activation markers
AUC: Area under the curve
CTLA-4: Cytotoxic T lymphocyte associated protein 4
EPP: Events per parameter
FFPE: Formalin-fixed paraffin-embedded
FMO: Fluorescence minus one
GC: Gastric cancer
HDs: Healthy donors
H&E: Hematoxylin–eosin
HR: Hazard ratio
IMs: Inhibitory molecules
IHC: Immunohistochemistry
LAG-3: Lymphocyte-activation gene 3
MDSCs: Myeloid-derived suppressor cells
NSCLC: Non-small-cell lung cancer
OS: Overall survival
PBLs: Peripheral blood lymphocytes
PD-1: Programmed cell death-1
ROC: Receiver operating characteristic
T_N_: Naïve T cell
T_CM_: Central memory T cell
T_EM_: Effector memory T cell
T_TM_: Transitional memory T cell
T_EMRA_: Effector memory T cell re-expressing CD45RA
TME: Tumor microenvironment
TILs: Tumor infiltrating lymphocytes
TIM-3: T-cell immunoglobulin and mucin domain-3
TIME: Tumor immune microenvironment
