# Supplementary figures and images for: Identification and validation of a novel survival prediction model based on the T-cell phenotype in the tumor immune microenvironment and peripheral blood for gastric cancer prognosis
Source: J Transl Med. 2023 Feb 3;21:73. doi: 10.1186/s12967-023-03922-0 (PMC9896795; doi:10.1186/s12967-023-03922-0)

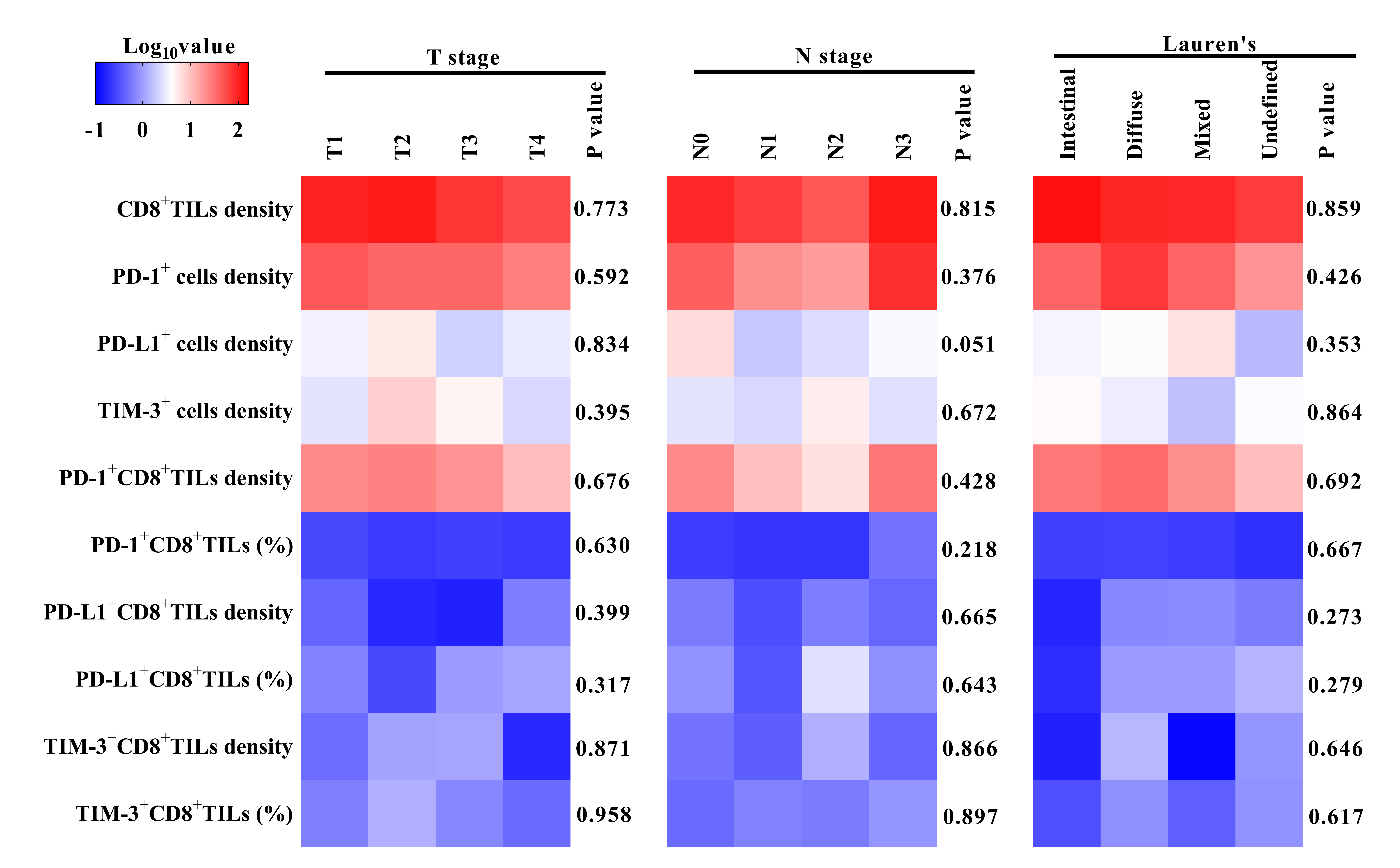

Supplement: Supplementary file 1 — Additional file 1: Figure S1. The difference of the density of CD8+TILs, PD-1+cells, PD-L1+cells, TIM-3+cells, PD-1+CD8+TILs, PD-L1+CD8+TILs and TIM-3+CD8+TILs, and the difference of the percentage of PD-1+CD8+TILs, PD-L1+CD8+TILs and TIM-3+CD8+TILs were analyzed based on the T stage, N stage and Lauren's classification. [file 12967_2023_3922_MOESM1_ESM.tif]

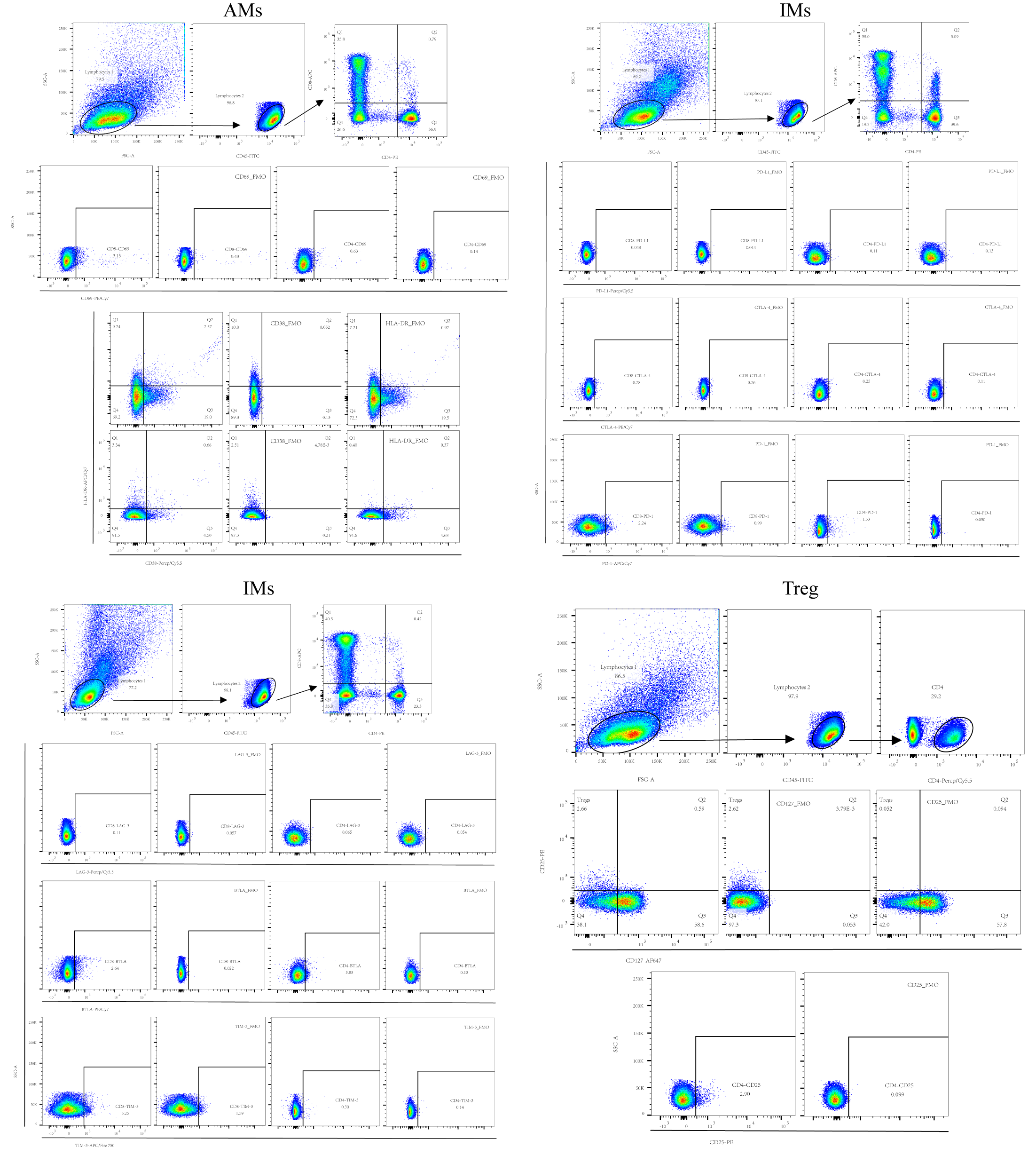

Supplement: Supplementary file 2 — Additional file 2: Figure S2. The analysis diagram of AMs, IMs, and Treg in PBLs. [file 12967_2023_3922_MOESM2_ESM.tif]

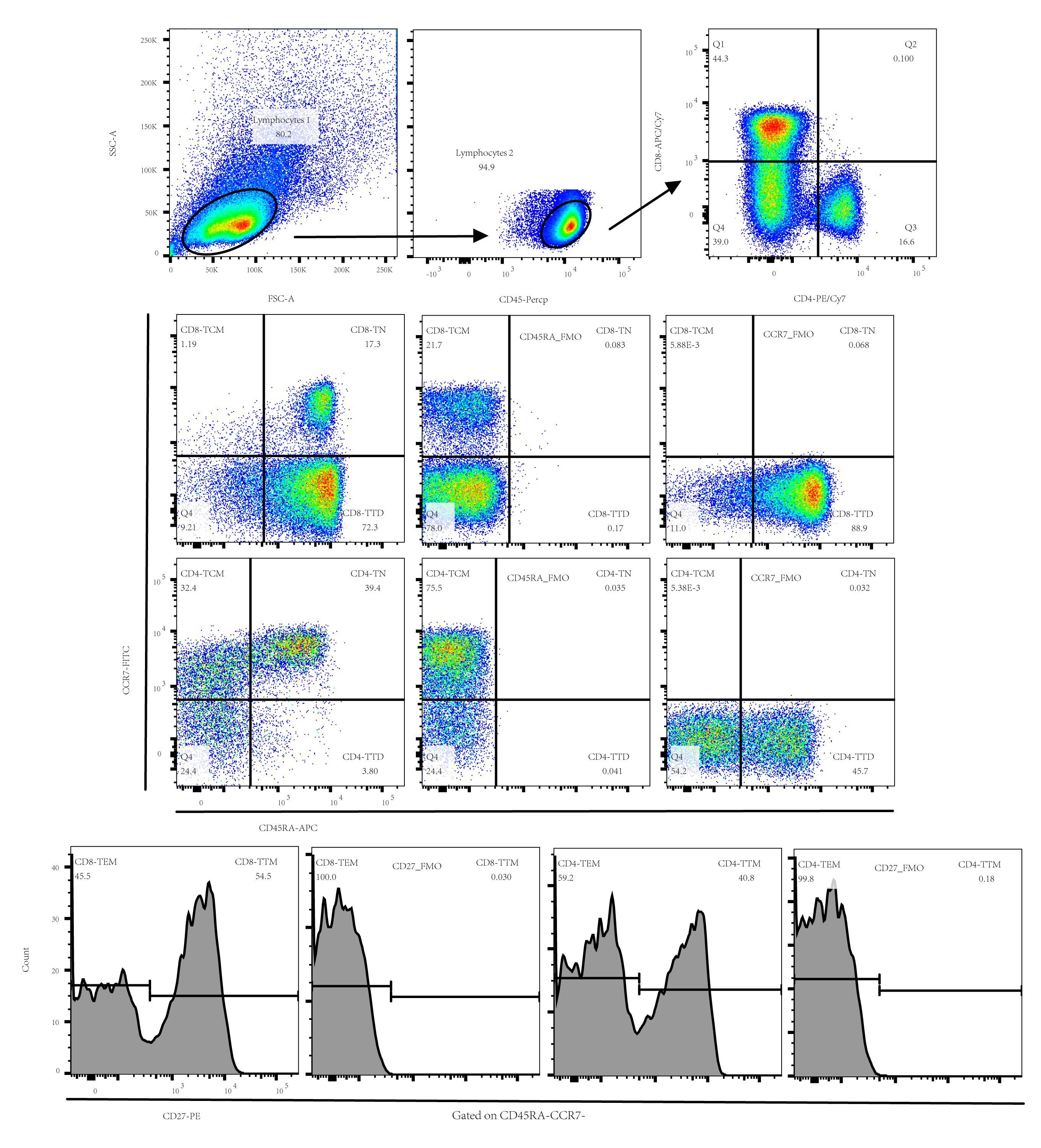

Supplement: Supplementary file 3 — Additional file 3: Figure S3. The analysis diagram of T cell subsets in PBLs. [file 12967_2023_3922_MOESM3_ESM.tif]

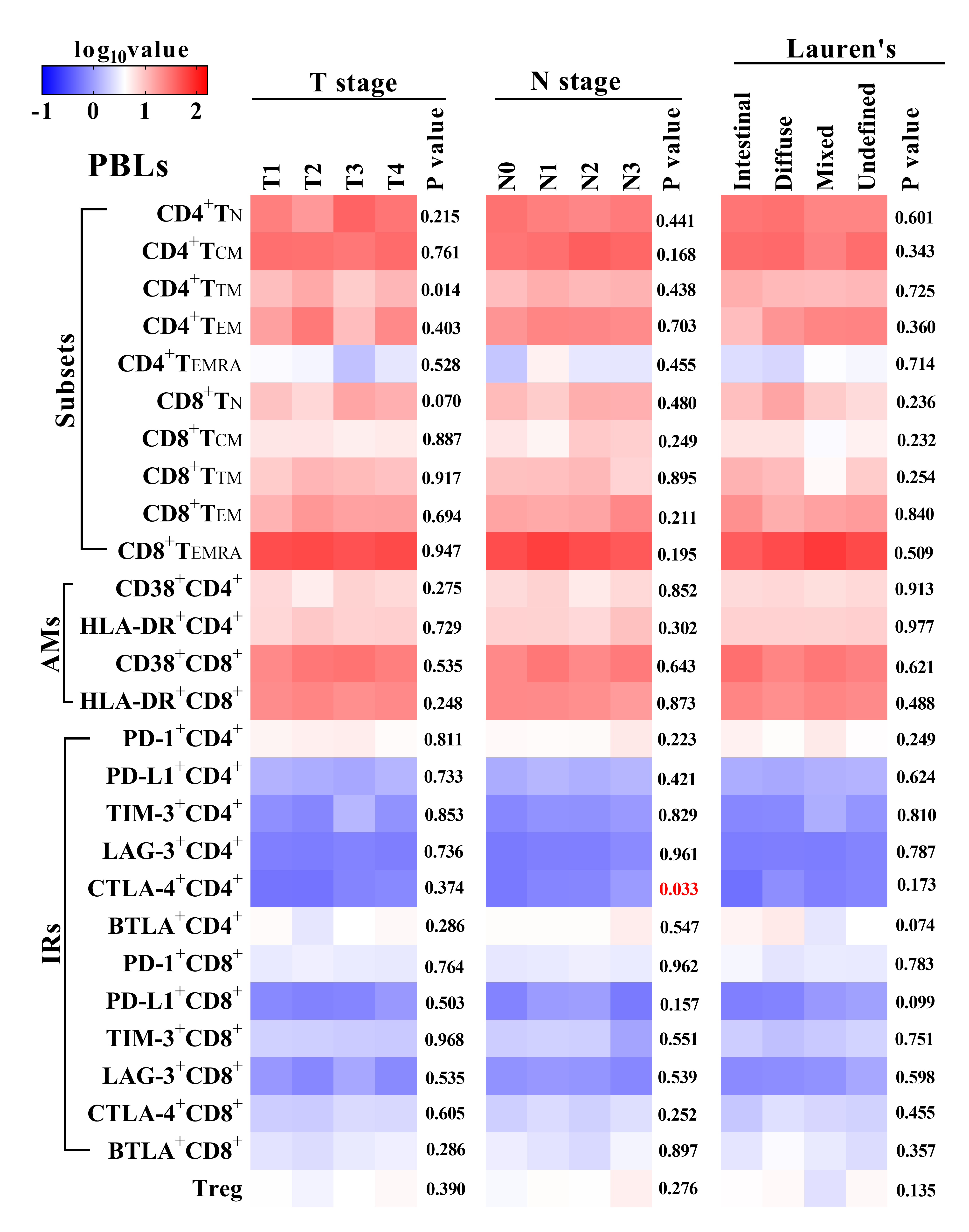

Supplement: Supplementary file 4 — Additional file 4: Figure S4. The T cell subsets distribution, AMs and IMs expression on T cells in PBLs were analyzed based on the T stage, N stage and Lauren's classification. [file 12967_2023_3922_MOESM4_ESM.tif]

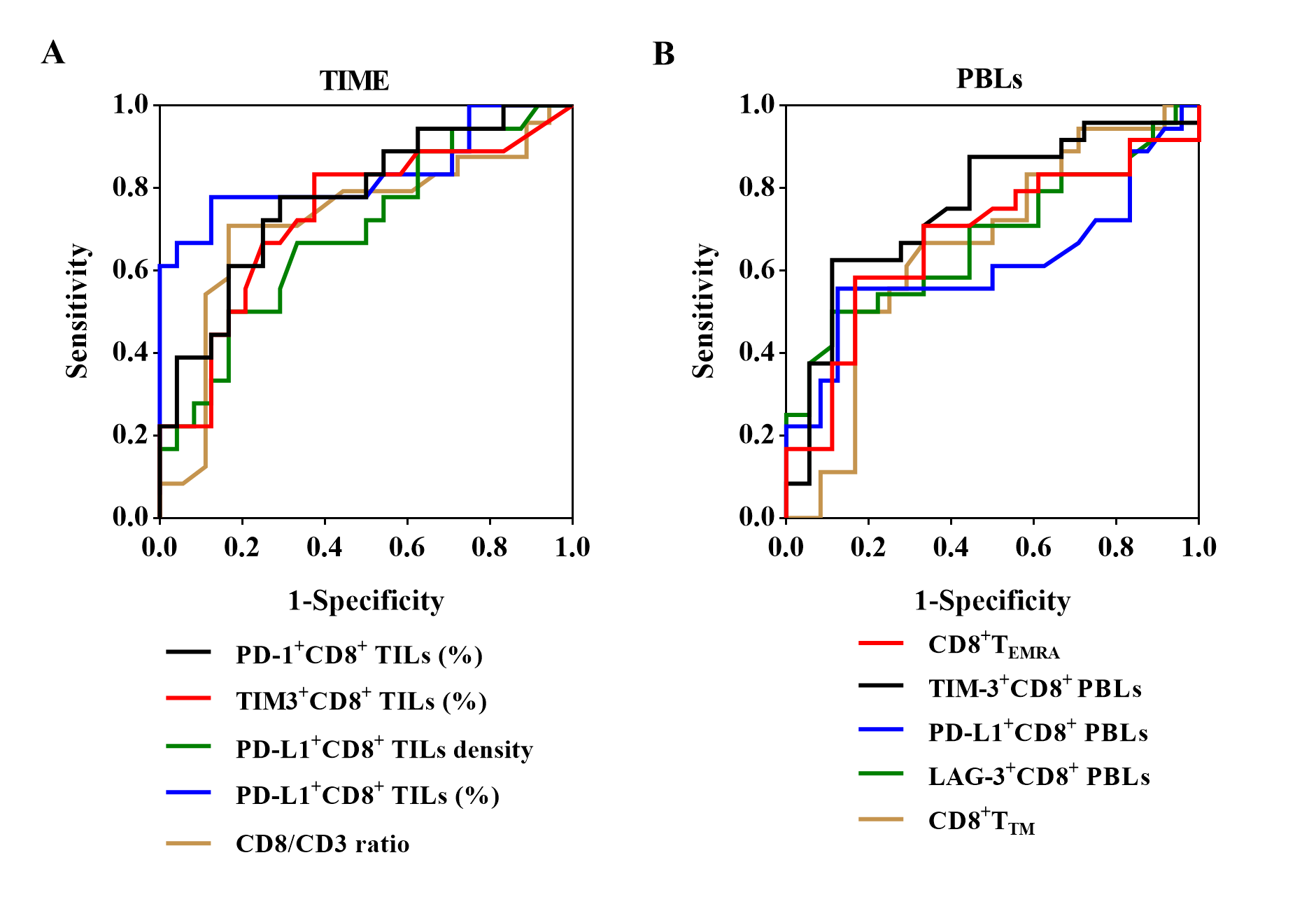

Supplement: Supplementary file 5 — Additional file 5: Figure S5. The analysis of the predicted survival value by ROC curves. [file 12967_2023_3922_MOESM5_ESM.tif]
