# Supplementary material for: Identification and validation of a novel survival prediction model based on the T-cell phenotype in the tumor immune microenvironment and peripheral blood for gastric cancer prognosis
Source: J Transl Med. 2023 Feb 3;21:73. doi: 10.1186/s12967-023-03922-0 (PMC9896795; doi:10.1186/s12967-023-03922-0)
Supplement: Supplementary file 6 — Additional file 6: Table S1. The clinical-pathological characteristics of TIME-A and TIME-B. Table S2. The clinical pathological characteristics of PBL-A and PBL-B. Table S3. Sensitivity and specificity of TIME and PBLs parameters in predicting prognosis. Table S4. Multivariate Cox regression analysis for the candidate parameters of model 3 in the internal cohort. Table S5. The clinical pathological characters of patients. [file 12967_2023_3922_MOESM6_ESM.docx]

**Table S1 The clinical-pathological characteristics of TIME-A and** **TIME-B.**

|  | TIME-A | TIME-B | P value |
| --- | --- | --- | --- |
| Gender | 38 | 9 | 0.091 |
| Male/ Female | 30/8 | 4/5 |  |
| Age (mean) | 58 | 56 | 0.680 |
| Tumor location | 38 | 9 | 0.195 |
| Upper 1/3 | 10 (26.3%) | 4 (44.4%) |  |
| Middle 1/3 | 10 (26.3%) | 0 (0.0%) |  |
| Lower 1/3 | 18 (47.4%) | 5 (55.6%) |  |
| Differentiation | 37 | 9 | 0.303 |
| Low | 10 (27.0%) | 4 (44.4%) |  |
| Moderate | 10 (27.0%) | 0 (0.0%) |  |
| Low-moderate | 16 (43.3%) | 5 (55.6%) |  |
| Moderate-high | 1 (2.7%) | 0 (0.0%) |  |
| AJCC | 38 | 9 | 0.047 |
| ⅠA | 9 (23.7%) | 2 (22.2%) |  |
| ⅠB | 6 (15.8%) | 0 (0.0%) |  |
| ⅡA | 3 (7.9%) | 2 (22.2%) |  |
| ⅡB | 3 (7.9%) | 4 (44.4%) |  |
| ⅢA | 12 (31.6%) | 0 (0.0%) |  |
| ⅢB | 3 (7.9%) | 1 (11.1%) |  |
| ⅢC | 2 (5.3%) | 0 (0.0%) |  |
| Lauren’s classification | 32 | 9 | 0.093 |
| Intestinal | 11 (34.4%) | 0 (0.0%) |  |
| Diffuse | 7 (21.9%) | 4 (44.4%) |  |
| Mixed | 4 (12.5%) | 3(33.3%) |  |
| Undefined | 10 (31.3%) | 2 (22.2%) |  |
| Vascular invasion | 37 | 9 | 0.698 |
| Yes/no | 25/12 | 5/4 |  |
| Nerve invasion | 37 | 9 | 0.384 |
| Yes/no | 30/7 | 6/3 |  |

**Table S2 The clinical pathological characteristics of PBL-A and PBL-B.**

|  | PBL-A | PBL-B | P value |
| --- | --- | --- | --- |
| Gender | 36 | 11 | 0.974 |
| Male/ Female | 26/10 | 8/3 |  |
| Age (mean) | 57 | 57 | 0.837 |
| Tumor location | 36 | 11 | 0.911 |
| Upper 1/3 | 11 (30.6%) | 3 (27.3%) |  |
| Middle 1/3 | 8 (22.2%) | 2 (18.2%) |  |
| Lower 1/3 | 17 (47.2%) | 6 (54.5%) |  |
| Differentiation | 35 | 11 | 0.220 |
| Low | 12 (34.3%) | 2 (18.2%) |  |
| Moderate | 9 (25.7%) | 1 (9.1%) |  |
| Low-moderate | 13 (37.1%) | 8 (72.7%) |  |
| Moderate-high | 1 (2.9%) | 0 (0.0%) |  |
| AJCC | 36 | 11 | 0.053 |
| ⅠA | 5 (13.9%) | 6 (54.5%) |  |
| ⅠB | 6 (16.7%) | 0 (0.0%) |  |
| ⅡA | 4 (11.1%) | 1 (9.1%) |  |
| ⅡB | 7 (19.4%) | 0 (0.0%) |  |
| ⅢA | 8 (22.2%) | 3 (27.3%) |  |
| ⅢB | 4 (11.1%) | 0 (0.0%) |  |
| ⅢC | 2 (5.6%) | 0 (0.0%) |  |
| Lauren’s classification | 32 | 9 | **0.049** |
| Intestinal | 10 (31.2%) | 1 (11.1%) |  |
| Diffuse | 10 (31.2%) | 1 (11.1%) |  |
| Mixed | 6 (18.8%) | 1 (11.1%) |  |
| Undefined | 6 (18.8%) | 6 (66.7%) |  |
| Vascular invasion | 35 | 11 | 0.702 |
| Yes/no | 26/9 | 7/4 |  |
| Nerve invasion | 35 | 11 | **0.043** |
| Yes/no | 30/5 | 6/5 |  |

**Table S3 Sensitivity and specificity of TIME and PBLs parameters in predicting prognosis.**

|  | Sen | Spe | Cut-off | Area | Sig. | 95%CI |
| --- | --- | --- | --- | --- | --- | --- |
| **TIME** |  |  |  |  |  |  |
| PD-1^+^CD8^+^TILs (%) | 0.778 | 0.708 | 23.70 | 0.775 | 0.002 | 0.632-0.919 |
| TIM-3^+^CD8^+^TILs (%) | 0.667 | 0.750 | 0.85 | 0.731 | 0.011 | 0.571-0.891 |
| PD-L1^+^CD8^+^TILs density | 0.500 | 0.833 | 0.84 | 0.691 | 0.036 | 0.529-0.853 |
| PD-L1^+^CD8^+^TILs (%) | 0.778 | 0.875 | 1.00 | 0.832 | <0.001 | 0.693-0.971 |
| CD8/CD3 ratio | 0.708 | 0.833 | 0.48 | 0.721 | 0.015 | 0.557-0.885 |
| **PBLs** |  |  |  |  |  |  |
| CD8^+^T_EMRA_ (%) | 0.583 | 0.833 | 56.05 | 0.679 | 0.049 | 0.514-0.845 |
| TIM-3^+^CD8^+^PILs (%) | 0.625 | 0.889 | 2.03 | 0.760 | 0.004 | 0.611-0.910 |
| PD-L1^+^CD8^+^PILs (%) | 0.556 | 0.875 | 1.03 | 0.616 | 0.204 | 0.427-0.804 |
| LAG-3^+^CD8^+^PILs (%) | 0.500 | 0.889 | 1.04 | 0.676 | 0.053 | 0.514-0.838 |
| CD8^+^T_TM_ (%) | 0.667 | 0.667 | 10.00 | 0.659 | 0.082 | 0.489-0.828 |

Sen: sensitivity, Spe: specificity.

**Table S4 Multivariate Cox regression analysis for the candidate parameters of model 3 in the internal cohort.**

|  | Multivariate analysis | |
| --- | --- | --- |
|  | HR (95%CI) | P |
| N status | 1.107 (0.727-1.686) | 0.637 |
| PD-1^+^CD8^+^TILs (%) | 6.989 (2.036-23.989) | 0.002 |
| PD-L1^+^CD8^+^TILs (%) | 17.105 (3.318-88.168) | 0.001 |
| PD-L1^+^CD8^+^PBLs (%) | 0.869 (0.247-3.053) | 0.826 |
| TIM-3^+^CD8^+^PBLs (%) | 0.159 (0.39-0.651) | 0.011 |

**Table S5 The clinical pathological characters of patients.**

|  | Internal cohort (n=47) | External cohort (n=70) |
| --- | --- | --- |
| Gender |  |  |
| Male/ Female | 34/13 | 55/15 |
| Age (mean) | 57 | 55 |
| Tumor location |  |  |
| Upper 1/3 | 14 (29.8%) | 15 (21.4%) |
| Middle 1/3 | 10 (21.3%) | 18 (25.7%) |
| Lower 1/3 | 23 (48.9%) | 37 (52.9%) |
| Differentiation |  |  |
| Low | 14 (30.4%) | 27 (38.6%) |
| Moderate | 10 (21.7%) | 12 (17.1%) |
| High | 0 (0.0%) | 7 (10.0%) |
| Low-moderate | 21 (45.7%) | 16 (22.9%) |
| Moderate-high | 1 (2.2%) | 8 (11.4%) |
| AJCC |  |  |
| ⅠA | 11 (23.4%) | 7 (10.0%) |
| ⅠB | 6 (12.8%) | 9 (12.9%) |
| ⅡA | 5 (10.6%) | 10 (14.3%) |
| ⅡB | 7 (14.9%) | 12 (17.1%) |
| ⅢA | 12 (25.5%) | 13 (18.6%) |
| ⅢB | 4 (8.5%) | 11 (15.7%) |
| ⅢC | 2 (4.3%) | 8 (11.4%) |
| Lauren’s classification |  |  |
| Intestinal | 11 (26.8%) | 21 (31.8%) |
| Diffuse | 11 (26.8%) | 17 (25.8%) |
| Mixed | 7 (17.1%) | 13 (19.7%) |
| Undefined | 12 (29.3%) | 15 (22.7%) |
| EBV status |  |  |
| Positive/negative | 5/42 | 3/67 |
| Vascular invasion |  |  |
| Yes/no | 30/16 | 50/20 |
| Nerve invasion |  |  |
| Yes/no | 36/10 | 43/27 |

**Figure S1**

The difference of the density of CD8^+^TILs, PD-1^+^cells, PD-L1^+^cells, TIM-3^+^cells, PD-1^+^CD8^+^TILs, PD-L1^+^CD8^+^TILs and TIM-3^+^CD8^+^TILs, and the difference of the percentage of PD-1^+^CD8^+^TILs, PD-L1^+^CD8^+^TILs and TIM-3^+^CD8^+^TILs were analyzed based on the T stage, N stage and Lauren's classification. Kruskal-Wallis H test was used to analyze the differences in T stage, N stage and Lauren’s classification, and the P value was shown on the right.

**Figure S2**

The analysis diagram of AMs, IMs, and Treg in PBLs.

**Figure S3**

The analysis diagram of T cell subsets in PBLs.

**Figure S4**

The T cell subsets distribution, AMs and IMs expression on T cells in PBLs were analyzed based on the T stage, N stage and Lauren's classification. Kruskal-Wallis H test was used to analyze the differences in T stage, N stage and Lauren’s classification, and the P value was displayed on the right. T cell subsets: T_N_, T_CM_, T_TM_, T_EM_ and T_EMRA_. AMs: active markers, CD38 and HLA-DR. IRs: inhibitory receptors, PD-1, TIM-3, LAG-3, BTLA, CTLA-4 and PD-L1. PBLs: peripheral blood lymphocytes.

**Figure S5**

The analysis of the predicted survival value by ROC curves. (A) ROC curves of PD-1^+^CD8^+^TILs percentage, TIM-3^+^CD8^+^TILs percentage, PD-L1^+^CD8^+^TILs percentage, PD-L1^+^CD8^+^TILs density and CD8 to CD3 ratio in predicting 3-year survival. (B) ROC curves of five parameters of PBLs in predicting 3-year prognosis, including CD8^+^T_EMRA_, TIM-3^+^CD8^+^PBLs, PD-L1^+^CD8^+^PBLs, LAG-3^+^CD8^+^PBLs and CD8^+^T_TM_ proportion. PBLs: peripheral blood lymphocytes. TILs: tumor infiltrating lymphocytes. ROC: receiver operating characteristic.
